# MK2 inhibitor reduces alkali burn-induced inflammation in rat cornea

**DOI:** 10.1038/srep28145

**Published:** 2016-06-22

**Authors:** Yanfeng Chen, Wenzhao Yang, Xiaobo Zhang, Shu Yang, Gao Peng, Ting Wu, Yueping Zhou, Caihong Huang, Peter S. Reinach, Wei Li, Zuguo Liu

**Affiliations:** 1 Eye Institute of Xiamen University, Fujian Provincial Key Laboratory of Ophthalmology and Visual Science, Xiamen, Fujian, China; 2 Department of Basic Medical Sciences, Cancer Research Center, Medical College, Xiamen University, Xiamen, China; 3 School of Ophthalmology and Optometry and Eye Hospital, Wenzhou Medical University, Wenzhou, Zhejiang, China; 4 State Key Laboratory Cultivation Base and Key Laboratory of Vision Science, Ministry of Health, People’s Republic of China.; 5 Zhejiang Provincial Key Laboratory of Ophthalmology and Optometry, Wenzhou, Zhejiang, China; 6 Affiliated Xiamen Eye Center of Xiamen University, Xiamen, Fujian, China

## Abstract

MK2 activation by p38 MAPK selectively induces inflammation in various diseases. We determined if a MK2 inhibitor (MK2i), improves cornea wound healing by inhibiting inflammation caused by burning rat corneas with alkali. Our study, for the first time, demonstrated that MK2i inhibited alkali burn-induced MK2 activation as well as rises in inflammation based on: a) blunting rises in inflammatory index, inflammatory cell infiltration, ED1^+^ macrophage and PMN^+^ neutrophil infiltration; b) suppressing IL-6 and IL-1β gene expression along with those of macrophage inflammatory protein-1α (MIP-1α), intercellular adhesion molecule-1 (ICAM-1) and vascular cell adhesion molecule-1 (VCAM-1); c) reducing angiogenic gene expression levels and neovascularization (NV) whereas anti-angiogenic PEDF levels increased. In addition, this study found that MK2i did not affect human corneal epithelial cell (HCEC) proliferation and migration and had no detectable side effects on ocular surface integrity. Taken together, MK2i selectively inhibited alkali burn-induced corneal inflammation by blocking MK2 activation, these effects have clinical relevance in the treatment of inflammation related ocular surface diseases.

Ocular chemical burns are a common trauma encountered worldwide particularly in the developing countries^1^. They are more frequently a cause of alkaline injury than exposure to injurious acids^2^. Caustic agents can readily penetrate into the anterior ocular surface and produce chronic inflammation and opacification resulting in severe and permanent visual impairment^3^. Even though keratoplasty is a viable therapeutic option, the success of this procedure depends on first resolving inflammation and neovascularization (NV) with drugs^4^. Currently, many of the drugs used for this purpose are somewhat problematic since they can have side effects and only provide symptomatic relief rather than target the mechanisms underlying inflammation and NV^4^,^5^. This limitation has prompted numerous studies to delineate mechanisms underlying the pathogenesis of chronic inflammation and NV.

Unrelenting and dysregulated corneal inflammation and NV, are common sequels of an alkali burn that can lead to persistent visual impairment and delay performing penetrating keratoplasty^4^,^6^. Alkali burns induce chemokine driven immune cell corneal infiltration accompanied by rises in pro-inflammatory cytokines levels^5^,^7^,^8^,^9^,^10^,^11^. In addition, the tenuous balance between pro-angiogenic and anti-angiogenic factors can be disrupted leading to corneal NV^12^. Thus, suppression of these maladaptive injury-induced responses is essential for reducing loses in corneal transparency and hastening wound healing. Various medical and surgical options such as steroids, nonsteroidal inflammatory agents, citrate, argon laser photocoagulation, and photodynamic therapy are used to treat corneal inflammation and inflammatory NV induced by an alkali burn^4^; however, sometimes, these therapies are ineffective, especially for large inflammatory NV^13^. The novel approaches under investigation to improve treatment of chemical burns include manipulating the inflammatory and angiogenesis-related factors by means of monoclonal antibodies, receptor modification, aptamers, and inhibitors of candidate inflammatory and/or angiogenesis pathways^6^,^11^,^13^. Even though some of these options look promising, each one of them can have side effects that limit their usefulness in restoring corneal transparency and optical properties needed for normal vision^4^,^13^.

Mitogen-activated protein kinase–activated protein kinase-2 (MAPKAPK2 or MK2) is an intracellular serine/threonine kinase substrate downstream from p38 mitogen-activated protein kinase (MAPK) and its activation by p38 is implicated in many inflammatory diseases including postoperative ileus, pancreatitis, atherosclerosis, rheumatoid arthritis and cancer^14^,^15^,^16^,^17^,^18^,^19^. Accordingly, it is an established drug target for treating many inflammatory diseases since its activation selectively induces the translation and increases stability of pro-inflammatory cytokine, chemokine and cell adhesion factor mRNA^20^,^21^,^22^,^23^. There are no reports describing a role for MK2 in mediating corneal inflammation.

We show here that alkali burning rat corneas induces MK2 activation, which in turn contributes to corneal inflammation and NV. These adverse effects compromising wound recovery of corneal transparency are mitigated through selectively suppressing MK2 activation with a MK2 inhibitor: MK2i, a cell-penetrating peptide inhibitor of MK2. Accordingly, MK2 is a viable drug target for treating corneal wounds caused by alkali exposure.

## Results

### Validation of MK2i mode of action

**Figure 1:**
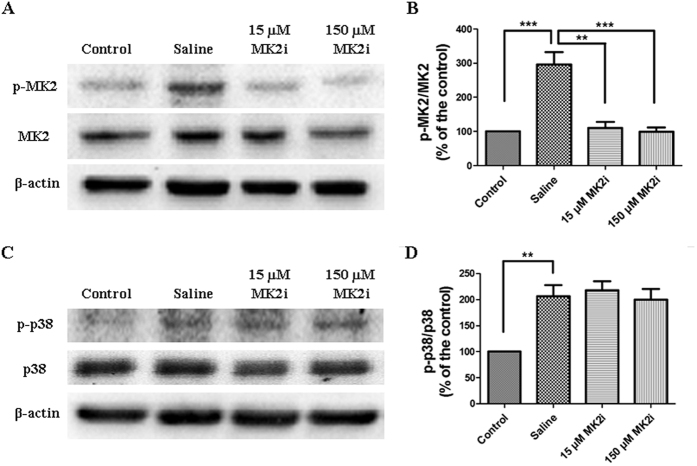
Selective suppression by MK2i of alkali burn-induced MK2 activation. **(A)** Representative Western blots images shows the selective inhibitory effects of MK2i on MK2 activation on day 7 after alkali burn; **(B)** Summary plot showing changes in the MK2 phosphorylation status of the 4 groups; **(C)** MK2i does not inhibit p38 phosphorylation status after 7 days; **(D)** Summary plot showing changes in the p38 phosphorylation status of the 4 groups. Applications of either 15 or 150 μM MK2i fully and selectively suppressed MK2 activation. (Data are presented as Mean ± SEM, n = 3. ^**^p < 0.01, ^***^P < 0.001).

Alkali exposure induced p38 MAPK and MK2 signaling activation was evaluated on day 7. Representative Western blots images of p38 and MK2 shown in Fig. 1A,C indicate that 15 and 150 μM MK2i fully and selectively inhibited an increase in MK2 phosphorylation status. On the other hand, saline treatment had no effect on either p38 or MK2 activation. The summary plots shown in Fig. 1B,D indicate that MK2 and p38 phosphorylation status increased 3-fold and 2-fold, respectively. In addition, with either 15 or 150 μM MK2i the p38 phosphorylation status was invariant whereas it completely blunted an increase in MK2 phosphorylation status.

### MK2i ameliorates alkali burn clinical signs

**Figure 2:**
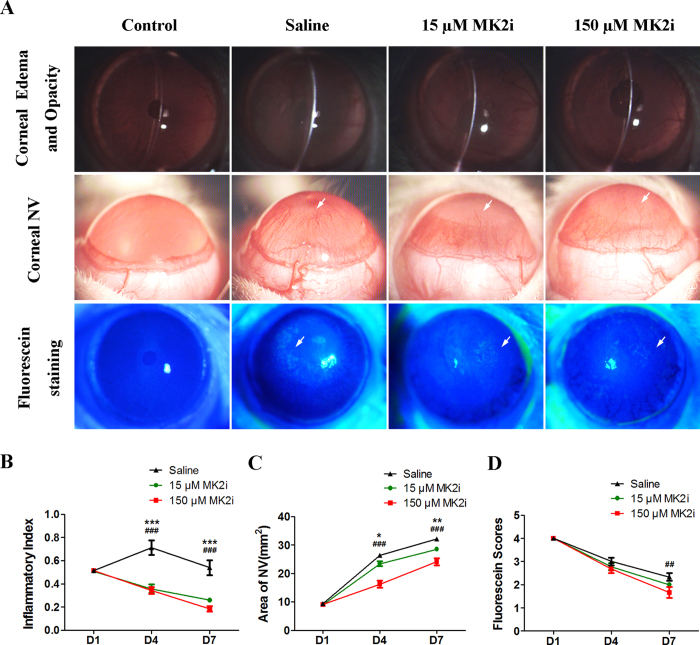
MK2i improves alkali burn clinical signs. (**A**) Representative slit lamp images showing differences between the 4 different groups in corneal edema, NV and fluorescein staining on day 7 after alkali burn; (**B**) Time dependent changes in inflammatory index among the 4 groups on days 4 and 7; (**C**) Time dependent changes in corneal NV area among the 4 groups on days 4 and 7; (**D**) Time dependent changes in fluorescein staining among the 4 groups on days 4 and 7. Topical MK2i treatment significantly decreased the corneal inflammatory index. Irrespective of the MK2i dose this inhibitor for the most part decreased clinical signs indicative of a corneal alkali burn. Only fluorescein staining decreased more by day 7 in the 150 μM MK2i treated group. (Data are presented as Mean ± SEM, n = 9. ^*^P < 0.05, ^**^P < 0.01, ^***^P < 0.001, 15 μM MK2i treated group versus saline treated group; ^##^P < 0.01, ^###^P < 0.001, 150 μM MK2i treated group versus saline treated group).

Alkali burning induced very apparent inflammation, NV and edema as well as opacification, which persisted up to day 7 (Fig. 2A). Compared to the saline treated group, both 15 and 150 μM MK2i decreased the day 4 and 7 inflammatory index (Fig. 2A,B). It initially rose in the alkali burnt group treated with saline before declining back to the baseline at day 1 (Fig. 2B). On the other hand, in the 15 and 150 μM MK2i treated groups the index fell continuously to a level below the baseline by day 7 (Fig. 2B). Obvious corneal NV was noted on day 7 in the saline treated group (Fig. 2A). NV increased less in the two MK2i treated groups than in the saline treated group (Fig. 2A,C). MK2i (150 μM) decreased the fluorescein staining score more than that caused by either 15 μM MK2i or saline (Fig. 2A,D), suggesting that the decrease in inflammation caused by 150 μM MK2i treatment promoted somewhat better corneal epithelial integrity recovery.

### MK2i suppresses immune cell infiltration

**Figure 3:**
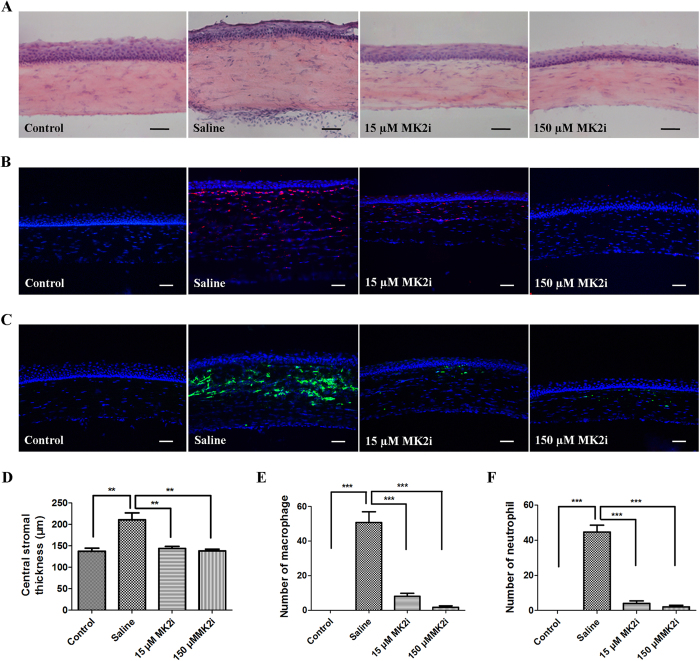
MK2i suppresses alkali burn-induced inflammation. (**A**) Representative H&E staining images on day 7 after burn. Topical MK2i treatment decreased corneal inflammatory cell infiltration and attenuated corneal thickening; (**B**) ED1 immunostaining on day 7; (**C**) PMN immunostaining was dramatically reduced by either 15 or 150 μM MK2i compared to the level obtained with saline treatment; (**D**) Summary plot of stromal thickness indicates either 15 or 150 μM MK2i decreased the thickness of the stroma; (**E**) Summary plot of stromal ED1^+^ macrophage infiltration indicates either 15 or 150 μM MK2i decreased corneal ED1^+^ macrophage infiltration; (**F**) Summary plot of stromal PMN^+^ neutrophil infiltration indicates the marked inhibitory effect of either 15 or 150 μM MK2i on this process. (Data are presented as Mean ± SEM, n = 3. ^**^P < 0.01, ^***^p < 0.001, Scale bars: 50 μm).

Histological analysis shows that both MK2i treatments completely blocked increases in stromal thickness caused by corneal alkali burning (Fig. 3A,D). Furthermore, epithelial and endothelial integrity was better preserved by MK2i treatment than in the saline treated group (Fig. 3A). Irrespective of its concentration, MK2i completely suppressed ED1^+^ macrophage and PMN^+^ neutrophil infiltration (Fig. 3B,C,E,F).

### MK2i suppresses pro-inflammatory cytokine and chemokine level rises

**Figure 4:**
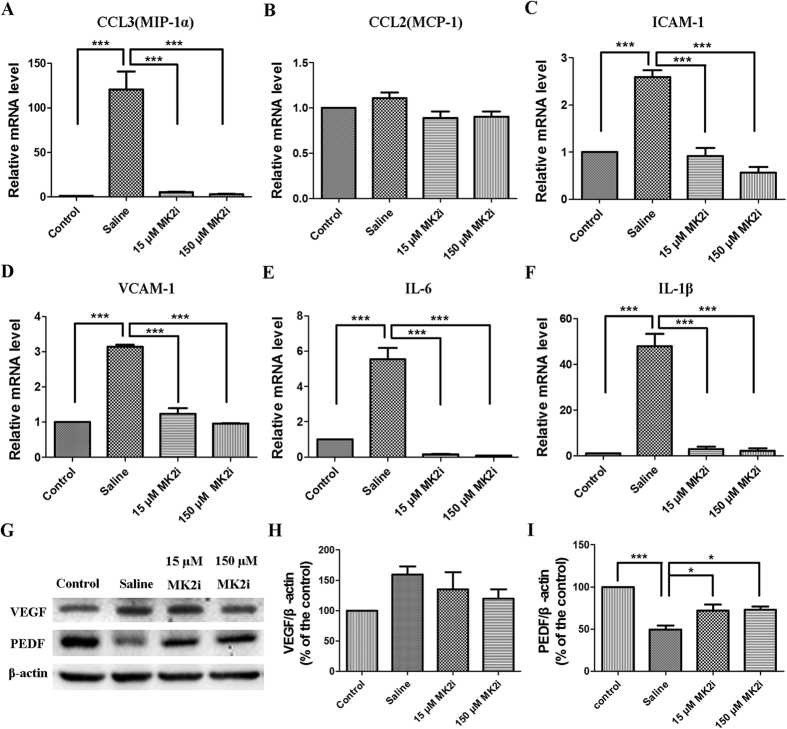
Modulation by MK2i of pro-inflammatory cytokine, chemokine, angiogenic and anti-angiogenic cytokine gene expression. Declines in mRNA levels induced by 15 and 150 μM MK2i of MIP-1α (**A**), ICAM-1 (**C**), VCAM-1 (**D**), IL-6 (**E**) and IL-1β (**F**) identified by qRT-PCR on day 7 whereas MCP-1 (**B**) remained unchanged; (**G**) Representative Western blot images and analysis of VEGF and PEDF protein expression levels on day 7 indicate that 150 μM MK2i somewhat decreased VEGF though no statistically significant difference was found compared with those in the saline treated group. PEDF decreased after alkali burns on day 7, while it partially recovered after MK2i treatment; (**H**) Summary plot of changes in VEGF protein expression induced by 15 and 150 μM MK2i on day 7 indicating that the difference was not statistically significant between each group; (**I**) Summary plot of changes in PEDF protein expression induced by low and high MK2i doses on day 7 indicating that with either 15 or 150 μM MK2i the protein expression level of this anti-angiogenic cytokine declined less than that in the saline treated control. (Data are presented as Mean ± SEM, n = 3. ^*^p < 0.05, ^***^p < 0.001).

Increases in chemokine, cell adhesion factors and pro-inflammatory cytokine levels contribute to inflammation and NV after an alkali corneal burn^5^,^7^,^8^,^9^,^10^,^11^,^24^,^25^,^26^,^27^. To determine if MK2 inhibition of corneal inflammation and NV is accompanied by suppression of these responses, we evaluated MK2i effects on representative angiogenic and pro-inflammatory cytokine gene expression levels on day 7. Both 15 and 150 μM MK2i very markedly decreased mRNA levels of a chemokine, MIP-1α, adhesion molecules ICAM-1 and VCAM-1 and pro-inflammatory cytokines IL-6 and IL-1β, while these two doses had no effect on MCP-1 chemoattractant expression mRNA (Fig. 4A–F). This inhibitor also had no effect on angiogenic VEGF protein expression whereas protein expression of the anti-angiogenic cytokine, PEDF, declined less during exposure to either 15 or 150 μM MK2i than in the saline treated group (Fig. 4G). Summary plots shown in Fig. 4H,I confirm that during MK2i treatment the VEGF level was unchanged whereas PEDF levels increased, respectively.

### Validation of MK2i response selectivity

**Figure 5:**
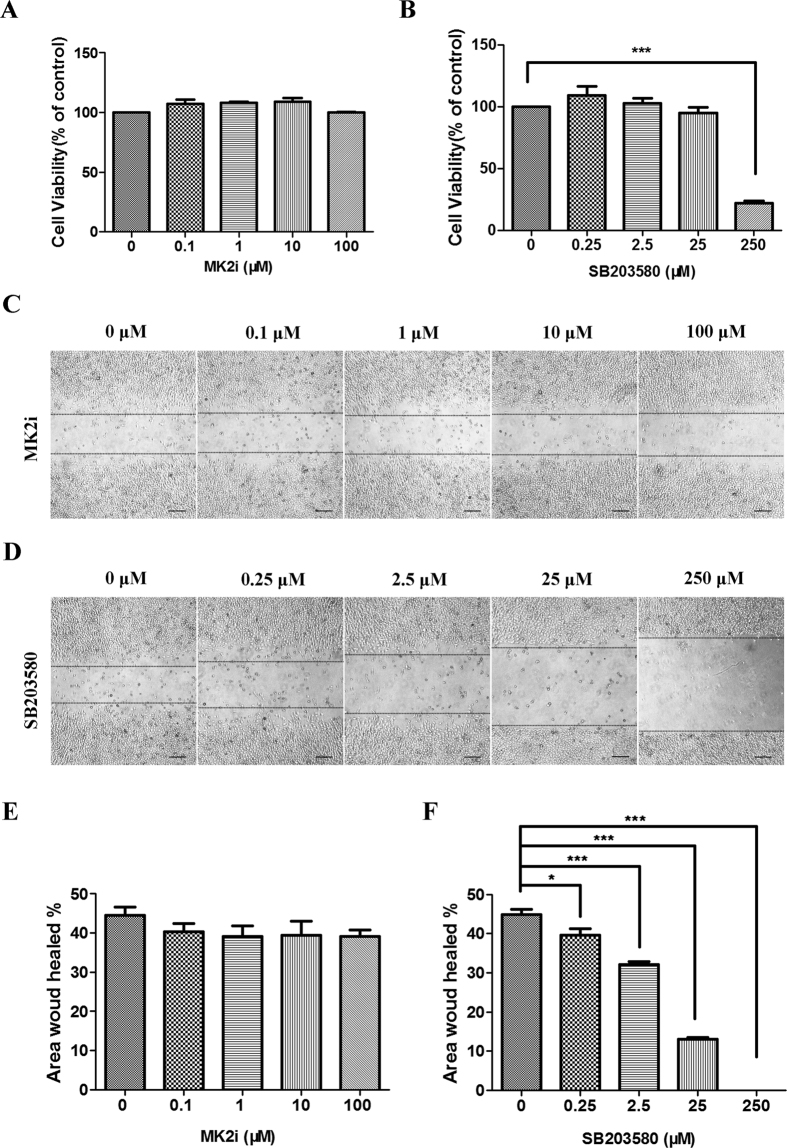
Failure of MK2i to inhibit HCEC proliferation and migration. (**A**) Irrespective of MK2i concentration MTS assay indicates after 24 h incubation that proliferation was unchanged; (**B**) SB203580 was used as a positive control to validate cell migration changes. The MTS proliferation assay shows that it became cytotoxic at 250 μM following 24 h incubation; (**C**) Representative images of HCEC wound closure at different MK2i concentrations. Wound closure was photographed 24 h after wounding; (**D**) Representative images of HCEC wound closure at different SB203580 concentrations. Wound closure was photographed 24 h after wounding; (**E**) Summary plots of the HCEC wound healing area expressed as a percentage 24 h after wounding in the presence of different MK2i concentrations; (**F**) Summary plots of the HCEC wound healing area expressed as a percentage 24 h after wounding in the presence of different SB203580 concentrations. (Data are presented as Mean ± SEM, n = 3. ^*^p < 0.05, ^***^p < 0.001, Scale bars: 200 μm).

To determine if the declines in inflammation and NV caused by MK2i are not associated with its inhibition of signaling mediators regulating cell proliferation and migration, we measured its effects on these two responses. These responses were chosen since they are mediated instead through increases in ERK1/2 and p38 MAPK activity, respectively, rather than MK2^28^,^29^. Figure 5A shows that none of the MK2i concentrations inhibited cell proliferation and they were not cytotoxic. A representative scratch wound assay shows that wound closure rate was unchanged regardless of the MK2i concentration (Fig. 5C). Figure 5E provides a summary of three experiments showing the insensitivity of HCEC migration to any MK2i concentration. With lower SB203580 concentrations, this selective p38 MAPK inhibitor did not decrease cell viability, but at 250 μM it was cytotoxic (Fig. 5B). On the other hand, as shown previously^28^, at lower SB203580 concentrations it progressively inhibited migration without affecting cell viability (Fig. 5B,D,F). For example, 25 μM SB203580 decreased cell migration by 71% without being cytotoxic. These results support the notion that the inhibitory effects of MK2i on inflammation and NV are limited to suppressing MK2 activation by p38 MAPK since MK2 does not modulate cell proliferation and migration^30^.

### Negative effects of MK2i on ocular surface health *in vivo*

**Figure 6:**
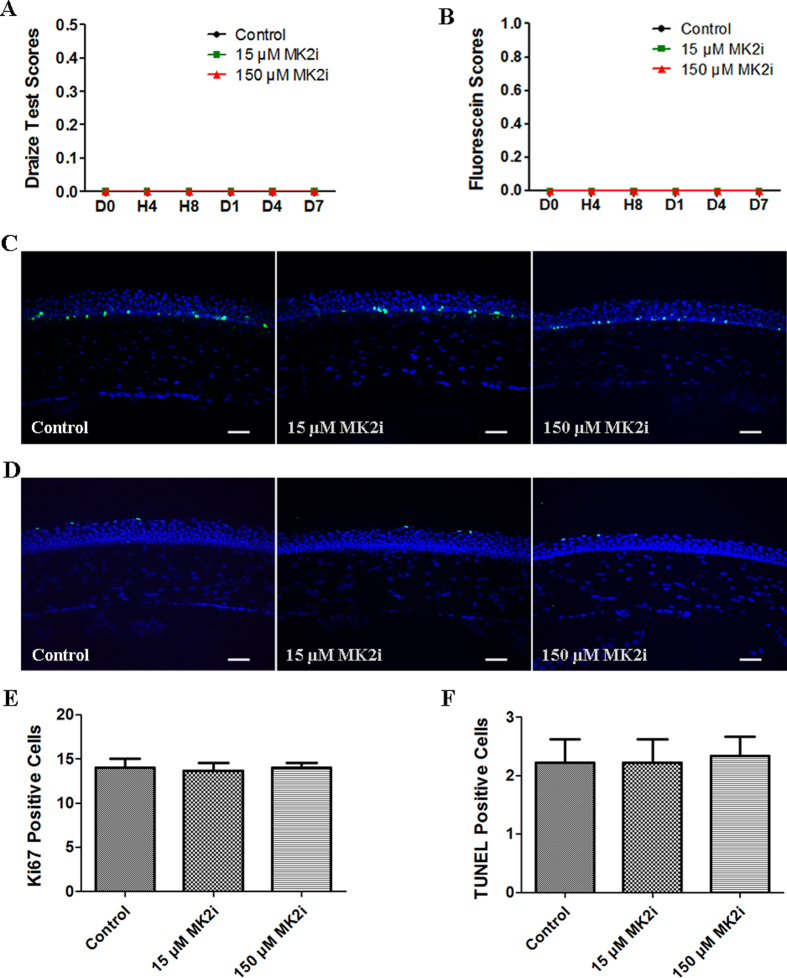
MK2i has no toxic effect on ocular surface. (**A**) Time dependent changes in Draize test scores among the three groups at 4 hour, 8 hour, 1 day, 4 day and 7 day (n = 6); (**B**) Time dependent changes in fluorescein staining among the 3 groups at 4 hour, 8 hour, 1 day, 4 day and 7 day (n = 6); **(C)** Representative images for immunofluorescent staining of Ki67 on day 7; (**D**) Representative images for TUNEL assay on day 7; (**E**) Summary plot of Ki67^+^ epithelial cells indicates either 15 or 150 μM MK2i did not inhibit corneal epithelial cell proliferation (n = 3); (**F**) Summary plot of TUNEL^+^ epithelial cells indicates either 15 or 150 μM MK2i did not inhibit corneal epithelial cell apoptosis (n = 3). (Data are presented as Mean ± SEM, Scale bars: 50 μm).

To determine if topical MK2i application induces any ocular surface defects, we used the Draize test and fluorescein staining to evaluate if this inhibitor induces ocular irritation and/or corneal barrier function disruption. It had no discernible effects on either ocular surface health or barrier function (Fig. 6A,B). To further assess if MK2i has other cytotoxic effects, we measured its effects on corneal epithelial cell proliferation and apoptosis. Accordingly, Ki67 immunofluorescent staining and TUNEL assay were performed on day 7, respectively. Only a few Ki67^+^ and TUNEL^+^ epithelial cells were detectable in all three groups, and no significant differences were found in the number of Ki67^+^ and TUNEL^+^ epithelial cells among the three groups (Fig. 6C–F). These results further substantiate that 15 and 150 μM MK2i do not compromise ocular surface health *in vivo*.

## Discussion

Corneal alkali burning induces inflammation and inflammatory NV as well as fibrosis. These effects lead to opacification and visual impairment, which cannot be corrected by performing keratoplastic surgery unless these responses to injury are first resolved by rendering them self-limiting^12^,^31^. As the therapeutic options provide essentially only symptomatic relief and are not very effective for reversing losses in visual acuity, additional insight is needed to identify novel drug targets for suppressing these side effects. This study is supportive of such efforts since it shows that MK2 inhibition by MK2i selectively reduced inflammation and led to NV suppression without having side effects that compromise re-epithelialization in rats.

p38 is involved in the pathogenesis of corneal inflammatory NV induced by either epithelial debridement or suture injury in animal models^32^,^33^. This signaling mediator directly induces MK2 phosphorylation, which in turn leads to pro-inflammatory mediator and chemokine gene expression in some tissues^34^. As p38 MAPK is also a component common to a variety of other intracellular signal transduction pathways mediating a host of different responses, selective p38 MAPK drug targeting does not selectively modulate inflammation. Its inhibition instead leads to increased liver enzyme levels and adverse central nervous system effects^35^,^36^. Accordingly, MK2 is a more viable drug target than p38 since its loss of function is not life threatening. As a matter of fact, MK2-deficient mice are healthy and have a normal phenotype while p38 knockout mice are embryonically lethal^14^. Accordingly, p38 MAPK inhibitor evaluation in clinical trials was discontinued because of their inhibition of responses promoting wound healing^28^,^35^,^36^. We now extend these studies by showing that MK2i selectively reduced inflammation and had effects that led to suppression of NV without inhibiting responses supporting corneal re-epithelialization. These effects of MK2i ought to prompt additional studies to determine if this peptide may be a lead compound for improving restoration of corneal transparency in other corneal wound healing animal models.

In response to injury, neutrophils followed by macrophages are recruited to an inflammatory site whereas macrophages remain there somewhat longer than neutrophils^37^,^38^. Robust increases in IL-1β gene expression occurred in the early injury stages and may be an initiator of the inflammatory response^27^. IL-6 also plays a significant role in corneal inflammatory disease as IL-6R antagonists reduce corneal inflammation and NV^11^. Chemokine MCP-1 and MIP-1α play central roles in neutrophil and macrophage recruitment^24^,^39^. Leukocyte adhesion molecules ICAM-1 and VCAM-1 facilitate leukocyte adhesion to activated vascular endothelial cells^10^. However, MK2i suppressed alkali burn-induced corneal inflammation based on declines in its inflammatory index, inflammatory cell infiltration and pro-inflammatory IL-1β, IL-6, MIP-1α, ICAM-1 and VCAM-1 cytokine gene expression along with elimination of massive stromal leukocyte infiltration.

Another finding consistent with declines in inflammation induced by MK2i is that this inhibitor reversed the disrupted balance between pro-angiogenic and anti-angiogenic factors by increasing PEDF levels. This cytokine is a potent anti-angiogenic factor which is localized to multiple ocular tissues including the RPE, retina, ciliary body, corneal endothelium, and epithelium^40^,^41^. Its anti-angiogenesis effect has been definitively demonstrated in numerous studies^42^,^43^,^44^. On the other hand, IL-1 and IL-6 are key pro-angiogenic factors^6^. ICAM-1-mediated leukocyte adhesion is a key event in early angiogenesis^45^. Increased ICAM-1 gene expression can lead to massive inflammatory cell infiltration, which in turn is a source of proangiogenic cytokines^10^. Accordingly, the increases in PEDF levels along with declines in representative pro-inflammatory cytokine and chemokine levels, indicate that part of the MK2i restorative effect stems from its reversal of the altered anti-angiogenic/angiogenic mediator ratio induced by injury. However, whether MK2i directly targets blood vessel endothelial cells needs to be further investigated.

To determine if the improved corneal wound healing outcome obtained with MK2i treatment is attributable to selective suppression of inflammation, we evaluated its effect on HCEC proliferation and migration. MK2i did not inhibit HCECs proliferation and migration and failed to compromise indicators reflective of ocular surface health *in vivo*. Since the nature of the corneal wound healing response following injury *in vivo* is dependent on the extent of damage to the limbal stem cell population^46^, it will be important to determine if MK2i treatment does not reduce this pool of cells giving rise to the differentiated epithelium.

In summary, this study shows that MK2i inhibited immune cell infiltration, stromal thickening, as well as declines of corneal epithelial and endothelial integrity in alkali burned corneas. MK2i may be a suitable compound to reduce inflammation in various ocular surface diseases.

## Materials and Methods

### Animals

Thirty-six male Sprague Dawley (SD) rats (180–200 g) were purchased from Shanghai Shilaike Laboratory Animal Co, Ltd., Shanghai, China. This study was performed in accordance with the ARVO Statement for the Use of Animals in Ophthalmic and Vision Research, and approved by the Experimental Animal Committee of Xiamen University (approval ID: XMUMC2013–03–3).

### Corneal alkali burn model

Rats were anesthetized with an intraperitoneal injection of 40 mg/kg pentobarbital and received topically a drop of tetracaine. A filter paper disc (3.5 mm in diameter) soaked with 1 N NaOH was then placed on the center of the corneal surface for 30 sec^47^,^48^. Following its removal, the ocular surface was rinsed with 10 ml saline.

### NMK2i treatment schedule

Rats were randomly divided into 4 groups: (1) control group that received no alkali burn; (2) corneal alkali burn group repeatedly washed with 10 μl saline 4 times per day for up to 7 days; (3) alkali burn followed by 10 μl of 15 μM MK2i (Millipore, Billerica, MA, USA), 4 times per day for up to 7 days; (4) alkali burn followed by10 μl of 150 μM MK2i (Millipore), 4 times per day for up to 7 days. All eyes were observed under a slit lamp microscope for baseline evaluation of corneal NV, inflammation, fluorescein staining and again 4 and 7 days later. All rats were thereafter euthanatized.

### Corneal NV evaluation

Images obtained under a slit lamp microscope were divided into 4 quadrants^47^. Vessel length of each quadrant (L_i_, i = 1–4) was measured with image analysis software (Image J; NIH, Bethesda, Maryland, USA). Vessel length was determined by measuring the perpendicular distances between the limbus and vessel tips, and the average vessel length in each quadrant was calculated. The corneal NV area (A) was calculated using the following equation: A = Σ_i=1−4_3.1416 × {R^2^ − (R − L_i_)^2^} (R is the radius of rat cornea. R = 3.5 mm, as determined in 36 rat corneas).

### Evaluation of corneal inflammation

Inflammatory index values were assigned based on the following evaluation: ciliary hyperemia (absent, 0; present but extending less than 1 mm, 1; hyperemia extending between 1 and 2 mm, 2; present and extending more than 2 mm, 3); central corneal edema (absent, 0; present with visible iris details, 1; present without visible iris details, 2; present without visible pupil, 3); and peripheral corneal edema (absent, 0; present with visible iris details, 1; present without visible iris details, 2; present with no visible iris, 3). The final inflammatory index result was obtained by summing the scores of the different parameters divided by 9^47^.

### Corneal epithelial damage assessment

Briefly, 5 μl of a 0.1% fluorescein sodium solution were instilled in a rat eye^47^. After 60 s, the eyes were examined with cobalt blue light under a slit lamp and photographed with a digital camera. The extent of corneal damage was scored according to the following scale: 0, no staining; 0.5, slight punctate staining; 1, diffuse punctate staining; 2, diffuse staining covering less than one third of the cornea; 3, diffuse staining covering more than one-third of the cornea; and 4, staining covering more than two-third of the epithelial layer area.

### Histology

After sacrifice on day 7, corneas were excised, embedded in optimal cutting temperature (OCT) compound (VWR, Suwanee, GA, USA), and flash frozen in liquid nitrogen. Sagittal 6 μm thick sections were cut with a cryostat (HM 500; Micron, Waldorf, Germany), placed on glass slides and stored at −80 °C.

### Staining procedures

Sections fixed in acetone at −20 °C were hematoxylin and eosin (H&E) stained. Light microscopy captured digital images of two representative areas of the central cornea (Nikon Eclipse 50i; Tokyo, Japan). Stromal thickness was measured using Image J Software (NIH) and its average thickness was obtained from triplicate slides per sample. Sections for immunofluorescent staining were fixed in acetone, blocked with 10% goat serum, and incubated with PMN primary antibody (1:4000, Fitzgerald, Newmarket Suffolk, United Kingdom), ED1 primary antibody (1:200, AbD Serotec, Oxford, United Kingdom) or Ki67 primary antibody (1:200, Abcam, San Francisco, CA, USA) at 4 °C overnight. Negative controls were performed at the same time by incubating a section with just PBS without any primary antibody. The next day, samples were incubated with Alexa Fluor 594-conjugated IgG (1:500, Invitrogen, Carlsbad, CA, USA) or Alexa Fluor488-conjugated IgG (1:500, Invitrogen) for 45 min in the dark at room temperature, followed by three washes in PBS. Nuclei were then counterstained using DAPI for 5 min. Digital images of three representative areas of the cornea were captured with a Leica microscope (DM2500; Leica Microsystems, Wetzlar, Germany). PMN^+^, ED1^+^ or Ki67^+^ cell averages were obtained from triplicate slides per sample.

### Western blot analysis

Rat corneal proteins were extracted with cold RIPA buffer. Aliquots having equal protein content were subjected to electrophoresis on 9% Tricine gels and then electrophorectially transferred to PVDF membranes. After 1 h blocking in 5% BSA, the blots were incubated with primary antibodies for: MK2(1:1000, Cell Signaling Technology, Danvers, MA, USA), Phospho-MK2(p-MK2, 1:1000, Cell Signaling Technology), p38(1:200, Santa Cruz Biotechnology Biotechnology, Dallas, TX, USA), Phospho-p38(p-p38, 1:1000, Cell Signaling Technology), PEDF(1:500, Abcam), VEGF(1:1000, Abcam) and HRP-conjugated anti-β-actin antibody (1:20,000, Sigma, Saint Louis, MO, USA) as a loading control. After washing each membrane three times with Tris-buffered saline containing 0.05% Tween 20 for 10 min, they were incubated with HRP-conjugated goat anti-rabbit IgG (1:10,000, Sigma) or HRP-conjugated goat anti-mouse IgG (1:10,000, Sigma) for 1 h at room temperature. The specific bands were visualized by an enhanced chemiluminescence reagent (ECL, Lulong Inc, Xiamen, China), and the image intensity was calculated with a transilluminator (ChemiDoc XRS System; Bio-Rad, Philadelphia, PA, USA).

### RNA isolation and qRT-PCR

Briefly, the corneas were cut into small pieces and homogenized in TRIzol^®^ (Invitrogen) on ice using a ULTRA-TURRAX^®^ digital homogenizer (IKA, Staufen, Germany). Total RNA of the corneas was extracted using TRIzol^®^ (Invitrogen) according to the manufacturer’s instructions. cDNA was synthesized using a reverse transcription kit (RR047A; TaKaRa, Shiga, Japan). Real-time PCR was performed on StepOneTM Real-Time PCR System (Applied Biosystems, Alameda, CA, USA) using a SYBR^®^ Premix Ex Taq Kit (RR420A; TaKaRa). In this study, Primer3 (http://primer3.ut.ee/) was used for designing primers, and their specificity was first verified by NCBI Primer Blast (http://blast.ncbi.nlm.nih.gov/Blast.cgi) (Table 1). The amplification program included an initial denaturation step at 95 °C for 10 min, followed by 40 cycles of 95 °C for 10 s, and 60 °C for 30 s after which a melt curve analysis was conducted to verify amplification specificity. Results were analyzed by the comparative threshold cycle (Ct) method, normalized with GAPDH as an endogenous reference and calibrated against the normal control group.

### Cell culture

A human corneal epithelial cell (HCEC) cell line was obtained from RIKEN Biosource Center (Tokyo, Japan) and cultured in supplemented hormonal epithelial medium (SHEM) containing DMEM-F12, 6% heat-inactivated fetal bovine serum, bovine insulin (5 μg/ml), recombinant human EGF (10 ng/ml) and 1% penicillin and streptomycin. Cultures were incubated at 37 °C under 95% humidity and 5% CO_2_.

### Cell proliferation assay

HCECs were seeded into 96-well plates at 5,000 cells/well, allowed to attach (4–6 h), and then either 0.1, 1, 10 or 100 μM MK2i. SB203580 (a pyridinyl imidazole inhibitor of p38 MAPK that specifically blocks its kinase activity) was present at either 0.25, 2.5, 25 or 250 μM(Cayman Chemical, Ann Arbor, MI, USA). After 24 h treatment, 20 μl of MTS solution provided in the CellTiter 96^®^ AQ_ueous_ One Solution Cell Proliferation Assay kit (Promega, Madison, WI, USA) were added to each well. Plates were incubated for an additional 2 h at 37 °C, after which the absorbance at 490 nm was recorded using a Bio Tek ELX800 microplate reader (Bio-Tek Instruments, Winooski, VT, USA) to calculate the cell survival percentages. Experiments were performed in triplicate and repeated three times.

### Cell migration assay

HCECs migration was evaluated after exposure to either SB203580 or MK2i. Cells were seeded into 12-well plates at a high density and grown until reaching confluence. The layers were then scratch-wounded using a sterile 1 ml pipette tip. The wells were washed two to three times with PBS to remove all loose or dead cells, and photographed (0 h). Then the medium was replaced with serum-free medium containing either 0.1, 1, 10 or 100 μM MK2i. SB203580 was present at either 0.25, 2.5, 25 or 250 μM. Control dishes were similarly scratch-wounded and incubated without addition of any inhibitor. After 24 h, each well was photographed at 4× magnification with a Nikon Te-2000u Eclipse epi-fluorescence microscope (Nikon). The extent of healing was measured using Image J Software (NIH). All the experiments were repeated three times, and the extent of healing is the mean of three experiments.

### Ocular surface toxicity evaluations

Eighteen male SD rats (180–200 g) were randomly divided into 3 groups: (1) control group with topical application of 10 μl saline 4 times per day for up to 7 days; (2) low MK2i dose group with topical application of 10 μl of 15 μM MK2i (Millipore), 4 times per day for up to 7 days; (3) high MK2i dose group with topical application of 10 μl of 150 μM MK2i (Millipore), 4 times per day for up to 7 days. The first instillation was chosen as time zero. All eyes were observed under a slit lamp microscope for baseline evaluation of ocular surface toxicity according to the Draize evaluation criteria^49^,^50^, fluorescein staining and again 4 hours, 8 hours, 1 days, 4 days and 7 days later. All rats were thereafter euthanatized. Corneas were excised, embedded in OCT compound (VWR), and flash frozen in liquid nitrogen. Sagittal 6 μm thick sections were cut with a cryostat (HM 500; Micron), placed on glass slides and stored at −80 °C for Ki67 immunofluorescent staining and terminal deoxynucleotidyl transferase mediated dUTP nick end labeling (TUNEL) assay.

### TUNEL assay

To measure end-stage apoptosis, *in situ* TUNEL assay was performed on frozen sections (DeadEnd Fluorometric TUNEL System G3250; Promega) according to manufacturer’s instructions. Sections were counterstained with DAPI (Vector, Burlingame, CA, USA), and digital images of three representative areas of the cornea were captured with a Leica microscope (DM2500; Leica Microsystems). The average number of TUNEL^+^ cells was obtained from triplicate slides per sample. For the positive control, sections were incubated in DNase I before the addition of equilibration buffer, while DDW was used instead of the TdT reaction mix in the negative control.

### Statistical analysis

Two-way analysis of variance test (ANOVA) with Bonferroni post hoc test was used to compare the differences in corneal NV, inflammation, epithelial damage and ocular surface toxicity among the groups. One-way ANOVA with a post hoc analysis Tukey test was conducted to analyze differences in Western blot, quantitative real-time PCR, immunofluorescent staining, TUNEL, proliferation and migration assay. Results were significant if P < 0.05.

## Additional Information

**How to cite this article**: Chen, Y. *et al*. MK2 inhibitor reduces alkali burn-induced inflammation in rat cornea. *Sci. Rep.* **6**, 28145; doi: 10.1038/srep28145 (2016).

## Figures and Tables

**Table 1:** Rat primer sequences used for qRT-PCR.

**Gene**	**Sense Primer**	**Antisense Primer**	PCR Product(bp)
IL-6	CACAAGTCCGGAGAGGAGAC	ACAGTGCATCATCGCTGTTC	168
IL-1β	CTGTGACTCGTGGGATGATG	GGGATTTTGTCGTTGCTTGT	210
intercellular adhesion molecule (ICAM-1)	ACGCAGTCCTCGGCTTCTG	GGTTCTTGCCCACCTGCTG	97
vascular cell adhesion molecule-1(VCAM-1)	ACAAAACGCTCGCTCAGATT	GTCCATGGTCAGAACGGACT	152
Ccl2(monocyte chemotactic protein-1 [MCP-1])	ATGCAGTTAATGCCCCACTC	TTCCTTATTGGGGTCAGCAC	167
Ccl3 (macrophage inflammatory protein-1α[MIP-1α])	TGCCCTTGCTGTTCTTCTCT	AAAGGCTGCTGGTCTCAAAA	152
GAPDH	GCAAGTTCAACGGCACAG	GCCAGTAGACTCCACGACAT	140
